# Protecting Canada’s Lab Animals: The Need for Legislation

**DOI:** 10.3390/ani12060770

**Published:** 2022-03-18

**Authors:** Vaughan Black, Andrew Fenton, Elisabeth H. Ormandy

**Affiliations:** 1 Schulich School of Law, Dalhousie University, 6061 University Avenue, Halifax, NS B3H 4R2, Canada; vaughan.black@dal.ca; 2 Department of Philosophy, Faculty of Arts and Social Sciences, Dalhousie University, Marion McCain Building, 6135 University Avenue, Halifax, NS B3H 4R2, Canada; atf@dal.ca; 3 Canadian Society for Humane Science, 300-225 West 8th Avenue, Vancouver, BC V5Y 1N3, Canada

**Keywords:** animal law, research ethics, animal experimentation, 3Rs, replacement, governance

## Abstract

**Simple Summary:**

In this paper we argue that the current oversight system for animal-based science in Canada needs major reform to keep pace with progressive legislation in other nations and to prioritize the replacement of animals as best scientific practice.

**Abstract:**

Canada’s current non-legislated oversight system for animal-based science not only fails to adequately incentivize the replacement of sentient animals as best scientific practice in any meaningful way, but also fails to adequately protect those animals bred, harmed, and killed in the name of science. In this paper, we outline the various shortcomings of the Canadian Council on Animal Care, and we highlight the need for Canada to move towards national legislation akin to that seen in other jurisdictions like the U.K. We conclude that while legislation alone cannot ensure the replacement of sentient animals in science, it appears to be a precondition for significant progress in animal protection and for the development and adoption of non-animal methods.

## 1. Introduction

Despite Canada having an oversight system for animal-based science that incorporates a commitment to the 3Rs (replacement, reduction, refinement [1]), in recent years the number of sentient animals subjected to scientific use in Canada has increased [2,3]. In part, that may be simply another feature of an animal welfare system in Canada that in other realms—agriculture, entertainment, hunting—purports to value animal well-being and high ethical standards, yet in its legal protections routinely falls short of those found in other industrialized countries. We argue here that Canada’s approach to animal welfare in the course of scientific use is particularly outdated and is in need of modernization. Furthermore, when it comes to animal-based science Canada has adopted an approach that sets numerous obstacles in the way of the replacement or reduction in animals used and has been particularly recalcitrant to change.

Canada is a rarity among industrialized countries in lacking nationwide legislation addressing the use of almost all animals in scientific activities (i.e., research, teaching, and testing). While Canada does have piecemeal legislation that indirectly speaks to the use of animals in specific scientific activities it has no equivalent to the provisions enacted by European Union countries in fulfillment of their obligations under the European Parliament’s directive on the use of animals for scientific purposes [4]. Nor does Canada have an equivalent to the U.S. *Animal Welfare Act* [5], the statute that governs experimentation on designated animals in the United States (or, for that matter, the “Common Rule” [6], which governs the use of humans in research in the U.S.). What is more, other industrialized countries like Australia, though lacking national legislation relevant to animal use in science, have state-level legislation across the country (and almost identical legislation across those states) [7]. Not all countries have a dedicated statute on the use of animals in scientific activities. For example, New Zealand has comprehensive legislation, the *Animal Welfare Act 1999*, but, that statute contains a chapter devoted to the regulation of animal use in research, testing, and teaching [8]. To be sure, animal law does not always provide adequate protections, and the jurisdictions we draw comparisons to have systems that differ markedly [9]. For example, despite its broad title, the U.S. *Animal Welfare Act* does not purport to address all aspects of animal welfare or even include all sentient animals. Among others, it excludes birds, mice, and rats as designated animals despite their common use in domains like science. Though this statute has been supplemented by the *Health Research Extension Act*, PL-99-158, 1985, its inadequacy remains. That said, such legislation makes designated animals visible to law and permits, even if only in principle, legal efforts to protect their interests.

This difference between Canada and other nations is not a recent occurrence. The United Kingdom passed dedicated animal protection legislation regarding scientific procedures in 1876 [10] and in 1986 it replaced that Victorian era act with a modernized statute, the *Animals (Scientific Procedures) Act 1986* (ASPA). The U.S. *Animal Welfare Act* is more than half a century old. It is true that Canada has had an animal cruelty provision in its criminal law since shortly after its 1867 formation—the offence is currently found in section 445.1 of the *Criminal Code* [11]. At least in theory, that provision might be brought to bear on situations of abuse or undue suffering of animals in the course of scientific activities. What is more, Canada’s recent *Ending the Captivity of Whales and Dolphins Act* [12] has two provisions that touch, albeit in passing, on research on captive cetaceans. However, neither of these features of Canadian law is a significant exception to the observation that Canada lacks national legislation on the protection of animals commonly used in science. Canada’s *Criminal Code* animal cruelty offences never address the specifics of biomedical and industrial animal-based experimentation, or animal-based scientific activities more generally. Its general provisions apply to maltreatment of animals that go beyond generally accepted practice in all settings—agriculture (terrestrial and aquatic), entertainment, hunting, companion animals, and so on. In practical terms, however, those provisions have little application to the use of animals in scientific activities. They are useful for addressing the actions of the intentionally cruel or neglectful but they are insufficiently fine-grained to be brought to bear in the context of routine yet harmful scientific use of animals; they are in no way comparable to the European and U.S. laws mentioned above. As a measure of this, it should be noted that no Canadian prosecutor has ever initiated a *Criminal Code* prosecution arising from harm to animals in the course of teaching or scientific experimentation. The same can be said with respect to provincial animal protection laws, which in theory apply to mistreatment of animals in the context of scientific use. It is our suspicion that they could not be successfully so brought to bear.

However, while Canada lacks a *legislated* regime dealing with the treatment of animals in the course of scientific study, regulatory testing, and science education, it nevertheless possesses an oversight agency that sets out standards applicable to those practices and can, in theory, administer sanctions to promote compliance. Since 1968, Canada has permitted the Canadian Council on Animal Care (CCAC) to administer guidelines regarding the scientific use of animals. The CCAC system enables Canada to claim that, like other industrialized nations, it responsibly protects animals’ welfare in the fields of scientific inquiry or training. An examination of that system reveals that it falls short.

## 2. The CCAC System

In the mid-1960s, stories in two popular U.S. magazines, *Life* and *Sports Illustrated*, offered exposés of animal suffering in the course of, or being bred for, scientific study. Those magazine stories prompted a public outcry that led the U.S. to pass legislation regulating the use of animals in science: the *Animal Welfare Act* [13]. Those magazines also circulated in Canada, which was not immune to public pressure to address concerns of undue animal suffering in scientific activities. Already, in November 1963, Canada’s Medical Research Council asked the National Research Council of Canada (NRC) to study the state of affairs regarding humane treatment of animals in scientific experimentation. The NRC struck a committee that then consulted with federal and provincial governments, universities, and other organizations, and in 1966 produced the *Report of the Special Committee on the Care of Experimental Animals* [14].

*The NRC Report* addressed the question of whether Canada should follow the U.S. and adopt research-specific animal protection legislation along the lines that the U.K. had introduced in 1876. Its response marked the point where Canada’s regulatory approach would diverge from that in the U.K. and U.S.:

“Contrary to some of the opinions expressed by the humane movement, the committee feels that the individual scientist will be influenced more effectively by his colleagues and peers working under a voluntary scheme than by legislation involving registration, licensing, and periodic visits by inspectors appointed under legislation.”

[14] (p. 27)

Instead of a command-and-control statute, the *NRC Report* recommended, at p. 29, a form of self-regulation through the establishment of a body of experts to be called the Canadian Council on Animal Care. This recommendation carried the day. Two years later the CCAC was established.

Although its terms of reference have changed marginally over the half century of its existence, in broad terms the CCAC oversight system remains much as was recommended in the 1966 *NRC Report*. The CCAC is a non-profit organization primarily funded by the Canadian Institutes of Health Research (CIHR) and the Natural Sciences and Engineering Research Council (NSERC), as well as by annual fees paid by members. Its member organizations include a number of industry representatives, such as the Canadian Association for Laboratory Animal Medicine, the Canadian Association for Laboratory Animal Science, and the National Farmed Animal Health and Welfare Council, government representatives like Agriculture and Agri-Food Canada, Defence Research and Development Canada, and Fisheries and Oceans Canada, as well as non-profits like the Canadian Cancer Society, Canadian Society of Zoologists, and Humane Canada [15]. Any institution receiving funding from CIHR or NSERC to conduct animal-based science is required to follow CCAC guidelines. Institutions that are not funded by CIHR or NSERC are entitled to seek CCAC certification but need not (unless otherwise specified by another funding agency). Those institutions under CCAC oversight must establish and maintain functioning animal care committees (ACCs) whose task is to undertake science ethics-guided review of proposals to conduct animal-based research, testing, or teaching. These local, institution-specific committees are charged with amending, approving, or rejecting research proposals and conducting follow-up monitoring of those protocols that are permitted to proceed. They are directed to make their decisions in accordance with guidelines established by the CCAC (which, at least in theory and to date, lean on the 3Rs of replacement, reduction, and refinement [1]) to ensure that the use of animal research subjects meets the standards the CCAC sets. In addition, the CCAC requires member institutions to provide appropriate veterinary care to animals under their control and further mandates that personnel working with animals have training in their care and handling. To support the acquisition of the relevant competencies, the CCAC provides training modules and other educational materials. As well as establishing governing principles and articulating specific guidelines, the CCAC conducts periodic, announced, inspections of member institutions to assess compliance. At least in principle, noncompliance can result in loss of eligibility for CIHR and NSERC funding. To the best of the authors’ knowledge this has only happened once since the establishment of the CCAC (data are not freely available to cite) despite annual Facts and Figures reports that show institutions are being put on probation every year (we assume this means they are noncompliant); for example, in 2020, of the 202 institutions under CCAC oversight, seven were put on probation (and assessment visits are not conducted for every institution every year) [16]. The CCAC also collects information regarding animal use in those institutions and makes some of those data public (for these and further details, see [17]).

## 3. Identifying the Problem

### 3.1. The Limits of a Voluntary System

When it was established in 1968, the CCAC approach—in particular, its innovation requiring local, institution-specific peer review committees—was perceived as a genuine advance [18]. At that time only a few countries had dedicated legal regulation of this field: the U.K. and U.S. have already been mentioned, to which should be added the Netherlands, Sweden, Germany, Norway, and Belgium. However, in 1968 many countries had no such legislation, and even some that did had oversight systems that were weak and porous.

By today’s standards, however, the CCAC system does not appear so forward-looking. The chief problem was present at the outset. Since participation in the CCAC system is voluntary for institutions not funded by CIHR or NSERC (or any other funding agency that requires CCAC certification), it will have no application to research institutions or companies (e.g., pharmaceutical corporations) that receive no such funding and do not elect to be governed by CCAC guidelines. Curiously, the fact that some research bodies might simply decline to join a voluntary organization was never mentioned in the NRC’s 1966 committee report. In retrospect that committee’s refusal to even take note of the possibility that some research bodies might decide not to embrace a scheme of voluntary self-regulation seems astounding. It is true that this defect is lessened slightly by the fact that all Canadian research universities are dependent on CIHR and NSERC funding. Accordingly, they have no real choice but to be certified by the CCAC and so submit to its oversight. However, this does not detract from the concern that the CCAC is (largely) irrelevant to non-certified institutions or companies using animals for scientific purposes in Canada, and it seems next to impossible to ascertain how many of these exist. As a result, the number and purpose of animals used in the private sphere in Canada is unknown (and unknowable), which is problematic for both oversight and public accountability. For these private entities there is no legal or other regulation of their use of animals apart from the (never invoked) *Criminal Code* and licensing legislation adopted by one of the ten provinces [19]. While this gap is troubling and on its own should itself be a reason for rethinking the 1968 decision to embrace a system based on voluntary self-regulation, there are other problems with the CCAC system.

### 3.2. Suboptimal Sanctions

Even for institutions that count on CIHR and NSERC funding (or any other agency that conditions funding on CCAC certification) and are thus effectively required to become CCAC members, available deterrents to incentivize compliance are suboptimal. For practical purposes the only “offence” in the system is if an institution commits “a material breach” of its obligations to comply with CCAC standards [20] (clauses 4.4 and 5.3(a)). Penalties for noncompliance with this vague standard are limited to the grants—suspending payment, requiring repayment, or declaring the institution ineligible to apply for future funding. These are significant penalties, but they are confined to the institution; they do not and cannot punish specific individuals, teams, or labs (e.g., by a fine or loss of license to conduct further animal-based research). What is more, the diffuse and, because of that, arguably unjust nature of these penalties works against applying them to whole institutions due to the misconduct of an individual or laboratory team. There is, for example, no possibility for the CCAC to levy a fine—as can be and is done under the U.S. *Animal Welfare Act* [21,22].

In the U.K., section 4 of the ASPA requires persons proposing to use animals in scientific procedures to have a personal license granted by the Secretary of State. This is in addition to the license that must be obtained for each individual project and the certificate that must be held by institutions in which such procedures or experiments take place. section 22 of the ASPA further provides that licenses may be suspended or revoked for failure to comply with the standards set out in the statute or the conditions in the license, and there is also the possibility of stronger sanctions—fines or imprisonment (for these and further details, see [23]). The CCAC regime permits none of this, so by comparison the incentive to remain compliant is much weaker than those seen in legislative systems.

We might briefly compare the current CCAC system with one where the Canadian government has turned to legislation to address concerns over animal suffering, however inadequate. Canada’s *Health of Animals Act* [24], and regulations made under it, address animal health and distress during transportation. These instruments bring into force both general prohibitions against causing unnecessary suffering during transport and specific rules about such matters as the length of time animals may be transported without stopping to give them a drink of water. These prohibitions are variously classified by degree of seriousness (i.e., mild, moderate, severe). The relevant legislated enforcement provides regulators a broad array of tools, ranging from warnings, compliance agreements, administrative monetary penalties (akin to traffic tickets), prosecution by summary conviction, and even proceeding by indictment, which carries the possibility of imprisonment, and moreover these enforcement mechanisms have been brought to bear e.g., [25]. The *Health of Animals Act* imposes liability both on individuals and institutions, with fines of up to $250,000, and provisions for the vicarious liability of employers. Statutory provisions calibrate the penalties by such factors as its seriousness, whether the offence was a first-time or subsequent one, whether it was carried out with a view to profit, and whether it was intentional or inadvertent.

In addition, a legislated enforcement system offers other advantages the CCAC system lacks: a clear process for bringing complaints to the relevant authorities, a more publicly accessible resolution process (including the possibility of a trial in open court and the stigma of a criminal record), and the possibility of privately initiated prosecutions in instances where authorities fail to act. In short, even for research institutions that do elect to become CCAC members—and it should never be forgotten that some do not—the unavailability of the sort of clearly defined prohibitions and calibrated, public deterrents that only legislation can provide renders the CCAC’s compliance incentives suboptimal if not ineffectual.

### 3.3. Inadequate Detection of Animal Welfare Problems

As noted, the CCAC conducts inspections of premises where scientific procedures on animals are carried out (if those premises belong to CCAC members). However, it does so only every couple of years and always gives many weeks’ notice of impending visits. This notice has advantages: participating institutions can ensure that the appropriate personnel are available to meet with inspectors, and the nature of the relationship between site visitors and facility personnel can be mostly collaborative and collegial in nature. However, it has obvious drawbacks as well: noncompliance between visits can be temporarily covered up so that it goes undetected. Since most animal research takes place in non-public spaces, this practice means there is little chance that undue animal suffering during scientific use in Canada will be discovered or exposed, especially as there is no legislative protection for whistleblowers. By way of contrast, the Preamble to the E.U. Directive requires member countries to provide for inspections and goes on to state:

“To ensure public confidence and promote transparency, an appropriate proportion of the inspections should be carried out without prior warning.”

[4] (Preamble, clause 36)

The U.K.’s ASPA—which was updated to conform to the E.U. Directive and remains in force post-Brexit—provides for inspection of premises holding licenses for experimenting on animals, and expressly notes that they may be carried out “without notice to the holder of the licence concerned” [23] (section 18, 2C). This is in addition to search and seizure powers given to the police in section 25 in respect of any cases where there are reasonable grounds to believe the Act is being violated. That license holders may be given no notice of a site visit by Home Office inspectors does not preclude a collaborative and constructive relationship between parties, but it does ensure that any missteps in ongoing welfare or other compliance issues are not readily covered up.

### 3.4. Lack of Transparency

Most scientific experimentation and testing involving animals takes place behind closed doors. CCAC members must provide the CCAC with written descriptions of those activities, but these are not publicly available or accessible. The CCAC publishes annual reports, which aggregate and make available some data it gathers, but they lack a specificity that would enable public accountability. Particularly lacking is any kind of detailed public reporting on noncompliance with CCAC guidelines: the reports that CCAC assessment directors make following their periodic review of member institutions are not public, or publicly accessible, documents. The only information provided by the CCAC is in brief Facts and Figures reports e.g., [16]. In contrast, the Home Office Animals in Science Regulation Unit (ASRU) in the U.K. publishes detailed compliance investigations [26], and annual reports that detail cases of noncompliance, and any action taken to rectify them [27]. Arguably, a “virtuous feedback” system for evaluation and quality assurance of animal research” [28], (p. 622) depends on such publicly accessible, evidence-based information.

The CCAC’s principles state that, in the interests of limiting animal suffering, scientists should attempt to reduce the number of animals they use. However, CCAC annual animal data reports only give numbers for the year in question; there is no indication given of trends over time. In contrast, U.K. and E.U. animal data reports give trends over time for total animal numbers (alongside notes of caution about comparing certain years if data collection strategies have changed), and the Home Office publishes non-technical but nevertheless detailed accounts of all animal research protocols that received approval [29]. Furthermore, neither the annual reports nor any other CCAC publication permits readers to tell whether a given CCAC member—a research university, for instance—is in fact reducing its animal use on a year-to-year basis or whether laboratory teams or educators are reducing animal numbers in their chosen areas of study or teaching. The annual animal data reports do not disclose data from individual institutions (which must be noted is also the case in the E.U. and U.K., and others have provided critiques of the data provided in the U.K. e.g., [30]).

Canada’s transparency problem is compounded by the already mentioned fact that participation in the CCAC is largely voluntary. To reiterate, since some research institutions elect not to be certified it is impossible to know the nature and full extent of animal experimentation in Canada. The CCAC collects and makes available some data from its members, but no one does the same from those research institutions that decline to join. Consequently, Canada is unable to supply accurate data on how many animals are used in scientific activities, what species are involved, or what the nature of the scientific use is. Furthermore, the CCAC itself is not subject to access to information legislation, so while transparency might be promised it is often not delivered.

### 3.5. Lack of National Uniformity

A further consequence of the lack of a nationwide legislative scheme is that even institutions that are CCAC certified and claim to follow its precepts and principles are not required to do so in a specified or uniform fashion. For example, universities purport to use their own forms and questionnaires to assess proposed research protocols for CCAC compliance [31]. This inhibits effective comparative study of how CCAC’s principles are in fact being administered. A study of the interpretation and application of ethical standards by members of animal ethics committees at four Canadian universities in different provinces showed considerable inconsistency in the understanding of those standards by committee members [32]. While it could be argued that lack of national uniformity is not unique to Canada, other jurisdictions with national legislation at least have harmonized legal standards—for example, while there are 50 states in the U.S., all with their own state-level legislation, the laws and guidelines that govern animal-based science are federal. National uniformity in legal standards helps prevent a “race to the bottom” dynamic across states (or provinces) and ensures that all institutions using animals for scientific purposes are subject to the same oversight (which, as we have highlighted in Section 3.1 above, is currently not the case in Canada).

### 3.6. Possibility of Unnecessary Duplication, Overuse, and Futile Research

The E.U. Directive [4] (article 37(1)(d) and Annex 6) provides that the protocol approval process should avoid the unnecessary duplication of procedures. In Canada, under the CCAC regime of dispersed, localized control, there is no required communication among either granting agencies or ACCs. Researchers at different institutions might be granted permission to undertake what is essentially the same experiment, resulting in significantly increased animal use with no gain in scientific knowledge. Of course, sometimes having the same experiment performed more than once has advantages; replicability is important. However, well-designed replication studies require a sensitivity to internal validity and that requires more than an accidental convergence of studies on a research question or method of study [33]. Where duplication occurs, it can waste time, money and, of particular interest in this context, animal lives. What is more, a lack of centralized detailed data on animal use in Canada slows down the ability of scientists to discern scientific dead-ends or futile avenues of research, which again wastes time, money, and animal lives. This can expand harms to include members of Canadian society who might benefit from more successful scientific pursuits using alternatives to what are in fact dead-ends or futile avenues of research [34]. Reduction in animal use is one of the CCAC’s stated goals, yet its dispersed structure and lack of centralized command inhibit its ability to achieve this.

### 3.7. Barriers to Effective Ethics Review

The CCAC-dependent regime does not require any kind of ethics assessment of proposed animal use until it is examined by ACCs at local institutions—and even at that point there is no place in the system where a true ethical review is conducted. As acknowledged by Laura Janara,

“…the CCAC claims that prevailing university subjections of animals provide “benefits” to humans that outweigh “costs” to those subjected…even if we entertain the CCAC’s philosophically problematic formulation of how to determine right or good, no comprehensive charting and analysis of such costs and benefits has ever happened in Canada, including as part of the oversight system. The sides of the ledger are not brought to bear on one another to demonstrate that the regime fulfils this justifying claim…”

[35] (p. 656)

Though we are restricted by our past or present service for the CCAC in what we can say about our own impressions of how ethics review is performed by ACCs, several things can reasonably be claimed in light of some published studies or guidelines (e.g., [36,37]). It is important to note that at least some of these issues will not be unique to Canadian ACCs (e.g., [38,39]). In practice, granting agencies review applications for their social value or scientific merit, and ACCs review for harms to animals. These two parties are not required to interact to do a true weighing of harms against benefits. A harm/benefit analysis of the sort mandated in the E.U. Directive and the U.K.’s ASPA is effectively structurally precluded in the Canadian system because by the time a research proposal reaches an institution’s ACC it has already been indicated (by the relevant funding body) that the proposed project has scientific merit. The ACC can then investigate whether the proposed use of animals is indeed required, whether fewer animals may be used, or whether refinements may be introduced to lessen suffering. However, as it cannot readily inquire into *how much* scientific merit the proposal enjoys, unless the relevant expertise is available in the committee or the greater institution and the relevant available experts are willing to make such a judgement, an ACC may be unable to undertake an approach that seeks to assess whether the animal suffering or harm implicated in a proposed study is outweighed by expected or sought-after benefits. The assessment of the quality of the research, its importance, and the benefits it is likely to achieve, relative to the known costs to the animals used, are integral to an ethics assessment [40]. Bad or trivial science is not ethical science. However, arguably, that a proposed study already enjoys financial support adds considerable pressure not to reject it on ethical grounds. If such a study coheres with past institutional practice or parallels already sanctioned scientific and even social use of animals (think of studies of intensive animal farming), it is reasonable to think that an ACC will be under considerable pressure to approve it. It is noteworthy that outright rejections of proposed animal use reviewed by ACCs are quite rare [37].

A full discussion of the inadequacy of relying on harm/benefit analysis in the context of animal-based science is outside the scope of this paper, however, it is the authors’ view that sole reliance on harm/benefit evaluation falls significantly short of adequate ethics review, and harm/benefit analysis has demonstrably fallen short of offering meaningful protection to animals used for scientific purposes, even in countries where harm/benefit analysis is legally required (e.g., [41]). While harm/benefit analysis has an important place in a full ethics review, we need only look to the use of humans in research to see how it is supplemented with other, more robust ethical principles and commitments. Though possible harms and sought-for benefits have long been a part of human research ethics, they are tempered by commitments to and legal protections of basic human rights, core principles such as respect for persons, concern for welfare, and justice, and shared understandings of a variety of harms that might befall human subjects/participants, including dying. Canadian policy in human-based science also favors providing participants with the final word on their participation, including those unable to consent or assent (for these and further details see [42]). This cannot be said of animal research ethics, or our understanding of animal research subjects.

Furthermore, a discussion on the ethics of animal use in science is not exhausted by merely applying the 3Rs or concepts related to animal welfare [43,44,45]. In striking contrast to human research ethics, as yet there are no internationally accepted ethics principles governing the use of animals in science [46,47]. The 3Rs are the only principles that come close, though they do not address the moral legitimacy of animal use (they assume it), cannot limit severity of use, cannot prohibit convenience killing, nor can they prohibit the use of certain species (unless the animal fails as an adequate model) [17,46,48].

In our view, a full appraisal of the ethics of animal use requires that we go beyond 3Rs considerations [49], otherwise other equally important ethical considerations may not even be raised in ACC meetings (e.g., the severity of the harm to the animals when the relevant use is normalized [regulatory testing, pain studies, vaccine research], whether the animals must be killed instead of rehomed, whether certain (or any) sentient animals should be used if they do not stand to benefit from their use and the risk of harm is beyond a minimal level, or whether killing is such a totalizing harm that it is difficult for any benefit to justify it) [48,49]. Not only should ACCs assess proposed animal use in deeper ways (consideration of deeper ethical questions is currently not required by ACCs), but to accomplish this these committees should not be limited to local institutions. Better compliance with the values of greater Canadian society requires that ethical assessments of proposed animal use happen at all levels, from the funding agencies deciding research priorities to the researchers seeking funding to pursue scientific activities. This requires a change in the oversight structure that is beyond the purview of the CCAC as it currently exists—we propose that a new legislated system for animal-based science in Canada be built on robust ethical principles that go beyond reliance on harm/benefit analysis and the 3Rs. Furthermore, a larger proportion of public representatives, and inclusion of ethics specialists, on ACCs would serve to better reflect the values of greater Canadian society [50,51].

### 3.8. Lack of Politically Responsible Oversight

The fact that there is no statute to make a given government ministry or department responsible for its administration means that Canadians have no politically responsible official or agency to which they can bring questions or concerns about the use of animals in scientific activities. Conceivably, a concerned party might approach the minister responsible for the CIHR or the one responsible for the NSERC, since those bodies are core CCAC funders. Alternatively, one could approach the minister for Agriculture and Agri-Foods or the minister of Fisheries and Oceans, since those two bodies are CCAC members, or (as has been done in the past) one could approach the Minister for Health or the Prime Minister’s Office. However, none of these officials takes direct political responsibility for CCAC policies and practices—as evidenced in the government letters provided in the Animal Alliance critique of the CCAC system [52] (pp. 126–128), in which government officials deflected questions simply by stating that the CCAC exists. Consequently, there is lack of clarity over who, if anyone, the CCAC is accountable to. So, although the public, through its tax dollars, contributes both to CCAC funding and to animal-based research conducted at institutions that CCAC oversees, there is no effective avenue of public accountability for this body or what is approved or regarded as legitimate animal use.

### 3.9. Disincentive to Replace and Reduce Animals in Scientific Activities

The CCAC’s dependency on the financial support of the NSERC and the CIHR limits its ability to effect substantive change in whether animals are used or how their use is assessed. Both the NSERC and the CIHR communities have vested interests in the continued use of animals in science and so are unlikely to support either more stringent assessments of animal use or a rejection of certain uses given the nature of the research or the preferred model species. That animal scientists themselves are deeply involved in the creation of the documents stating CCAC policies on animal use also compromises the ability of the CCAC to effect substantive changes in how animals are used or how their use is assessed, including moving beyond an ethics framework that is limited by favorable views of the 3Rs.

There is a growing critique of the efficacy and scientific validity of animal research models generally, especially against the increasing availability and reliability of in vitro and in silico methods (e.g., see [53]). Legislative oversight of animal-based research, with a provision on replacement, should act to stimulate government funding of reduction and replacement efforts. This bears out since the vast majority of progress that has been made to develop and validate new non-animal methods that replace harmful use of animals in science has been made in countries that have national legislation. For example, the E.U. Directive states that,

“…this Directive represents an important step towards achieving the final goal of full replacement of procedures on live animals for scientific and educational purposes as soon as it is scientifically possible to do so. To that end, it seeks to facilitate and promote the advancement of alternative approaches.”

[4] (Preamble, clause 10)

That this is written in the Directive that E.U. member states transpose into their national laws has meant that the E.U. has made significant progress on the development and implementation of non-animal methods through stimulation of research funding, roles for scientists in government to do research and write reports on non-animal approaches (e.g., [54,55,56]), and creation of progressive policies and other initiatives that promote the principles of replacement and reduction [57]. This is evidenced by the recent vote in the E.U. parliament where a resolution was adopted by 677 votes to 4 in favor of a faster phase out of animal research [58]. As another example, in 2016 the Netherlands made a commitment to end most regulatory animal testing by 2025 [59], and also created a Transition Programme for Innovation Without the Use of Animals [60]. Similarly, the U.K.’s ASPA has enshrined the development of “alternatives” as a legal requirement [22] (section 20B), and the U.K. Animals in Science Regulation Unit (ASRU) has published a plan with strategic goals to reduce the use of animals [61], along with corresponding delivery reports [62]. A major contribution to the ASRU delivery plan has been made by the National Centre for the Replacement, Reduction and Refinement of Animals in Research (NC3Rs) (a government-funded center). Meanwhile, in Canada, funding for the 3Rs program of the CCAC (which was established in 2008 [63]) was cut completely in 2012 when the CCAC lost two thirds of their grant funds from CIHR and NSERC. To date, there are no other national efforts in Canada to prioritize reduction and replacement of animal use in science.

U.K. annual animal statistics show annual decreases between 2015 and 2019 [64]. In 2020, animal numbers were lower still, though it is speculated by some experts that this decrease was due to the impact of COVID-19 and related restrictions [65]. Similarly, in the E.U. a 5% decrease in animal use was seen between 2017 and 2018, as per the latest data report [66]. The decrease in animal use seen in the U.K. and E.U. seems to correspond with implementation of the amended E.U. Directive in 2014; however, further research is needed to establish a causal relationship. In contrast, reported animal use in Canada has gone up in recent years, including in 2020, when the Canadian animal research community faced similar COVID-19 restrictions to the U.K. [2,3]. To be sure, the increase in animal use in Canada is not necessarily due to lack of legislation; however, in countries with legislation there appear to be substantial government funded efforts to advance non-animal methods, the likes of which are not seen in Canada. Key examples include the NC3Rs in the U.K. [67], the European Centre for the Validation of Alternatives Methods (ECVAM) in Europe [68], and the Interagency Coordinating Committee on the Validation of Alternative Methods (ICCVAM) in the U.S. [69]. In contrast, the Canadian Centre for Alternatives to Animal Methods (CCAAM) and its subsidiary, the Canadian Centre for the Validation of Alternative Methods (CaCVAM) [70] receive no government funding (outside of standard university research grants), and there are no other government funded efforts to reduce and replace animals in science.

Thus, having legal language that prioritizes non-animal approaches appears to incentivize government and private funding that creates a more competitive space for innovative non-animal methods in regulatory toxicology, both publicly funded and private research, and the use of animals in science education. We argue that such legal incentivization is needed in Canada.

## 4. The Need for Legislation

The bulk of the preceding critique of Canada’s approach to regulation of scientific use of animals has been on procedural defects: deficits of comprehensiveness, transparency, uniformity, accountability, and so on. That said we acknowledge that some of the shortcomings of the Canadian system are present in the legislated systems to which we draw comparisons; for example, to our knowledge, no jurisdiction provides animal use data at the institutional level, and the potential for unnecessary duplication of research is a global issue, not just an issue in Canada. We have not sought to focus on ways in which, in practice, the CCAC often falls short even of its modest oversight goals, though a recent report, *Between the Idea and the Reality* [52] provides convincing evidence that that is the case. Our focus on the formal structure of the CCAC system may seem removed from this volume’s focus on the substantive goal of replacing animal use in scientific exploration. However, we have sought to show how the agglomeration of procedural deficiencies—many of them inescapable features of a non-legislated, self-regulated scheme like the CCAC—operates to defer substantive progress, hamper public engagement, and curb reform that would prioritize the replacement of animals as best scientific practice. The *NRC Report* that led to the creation of the CCAC was a response to nascent, or at least apprehended, public concern with animal welfare in scientific activities. However, it took place without broad public participation and arrived at the solution that the problem should be resolved by letting scientists sort it out. The approach it selected—a non-profit, voluntary organization dominated by scientists who were animal users—not only lacked the procedural advantages of a legislated scheme but further perpetuated an arrangement where public input, especially as regards core values, remains difficult.

Considering the various ways the CCAC system falls short, we argue that national legislation would be the best direction in which the Canadian oversight system for animal-based science should evolve. To address the problems we have outlined with the current CCAC regime, a new legislative system should factor in the following elements:

- A clear, broadly supported, and regularly reviewed strategy to replace the use of animals in science in Canada, with interim timelines for specific activities (e.g., ending the use of animals in toxicity testing by 2035).
- Administration and oversight through a government department, such as the Ministry of Innovation, Science and Economic Development.
- Mandatory and uniform applicability to, and licensing of, all people and institutions who use animals in scientific activities.
- Clearly articulated ethical principles (that go beyond the 3Rs and harm/benefit analysis).
- Effective oversight and scrutiny, including proactive and randomized inspections that may occur without warning and gives authorization for inspectors to obtain search warrants where necessary.
- Central coordination of local review bodies to protect against unnecessary duplication of animal use.
- Enforceable and focused sanctions for noncompliance, including suspension of funds, loss of license, or prosecution of researchers, teams, or labs.
- Mandated annual reporting and publication of information and data on the nature and extent of animal use in scientific activities.
- Government funding for the development and implementation of alternative methods, and infrastructure that provides resources on how to implement non-animal methods.

While we argue here that the remedy, or at least precondition, for Canada’s obsolete oversight of scientific use of animals is a nationally applicable statute, it should be noted that the CCAC has taken the position that such action is not constitutionally permitted. Its website claims that only the provinces have legislative authority in this area, a situation which, were it true, would render modernization of this field far more difficult [71]. While there is no room here for a lengthy analysis of Canadian constitutional law, it should be noted that the CCAC’s position appears to ignore a longstanding and ongoing record of animal welfare legislation by the national government. We have noted three instances of this above: the animal cruelty provision in the *Criminal Code*, a federal statute dealing with animal welfare during transportation, and a statute ending the captivity of whales and dolphins that contains a specific provision dealing with research on those animals. The national government has passed other animal-protection laws as well: regulations promoting animal welfare in abattoirs and in the slaughter of seals, a statute protecting migratory birds, a statute promoting the welfare of species deemed to be at risk, and a criminal offence offering special protections to law enforcement and service animals. The case for legislative authority of Canada’s national government seems strong. Certainly, the provinces have legislative authority in this field as well, based on their power to regulate the use of property in the province, and we have noted above that one province has elected to exercise this power and has passed a statute regulating scientific use of animals in that province. However, the division of law-making power in Canada gives rise to many circumstances where both the central and the provincial governments may act, and in the event of a clash the national government’s federal laws take precedence.

It seems clear that before Canada can make progress toward significantly reducing and replacing the use of animals in scientific inquiry it needs to join other industrialized countries in adopting a legislated regulatory approach. Of course, the goal of replacing the use of animals in scientific inquiry in Canada is not guaranteed by legislative reform of the procedural defects of the CCAC system. However, such change appears to be a precondition to attaining that end.

## Data Availability

Data sharing not applicable.
